# IL-31—Pruritus Interleukin: Serum Values and Clinical Impact in Chronic Spontaneous Urticaria—A Romanian Retrospective Study

**DOI:** 10.3390/jcm12185957

**Published:** 2023-09-13

**Authors:** Carmen-Teodora Dobrican-Băruța, Diana Mihaela Deleanu, Ioana Adriana Muntean, Irena Pintea, Cristian Marius Florea, Gabriela Adriana Filip

**Affiliations:** 1 “Iuliu Hatieganu” University of Medicine and Pharmacy, Department of Allergology and Clinical Immunology, 400012 Cluj-Napoca, Romania; dobrican.carmen@umfcluj.ro (C.-T.D.-B.); diana.deleanu@umfcluj.ro (D.M.D.); 2 “Iuliu Hatieganu” University of Medicine and Pharmacy, Department of Cardiology, 400012 Cluj-Napoca, Romania; florea.cristian@umfcluj.ro; 3 “Iuliu Hatieganu” University of Medicine and Pharmacy, Department of Physiology, 400012 Cluj-Napoca, Romania; gabriela.filip@umfcluj.ro

**Keywords:** IL-31, CSU, UAS-7, pruritus, inflammation, atopy, IgE, eosinophils

## Abstract

(1) Background: This study aimed to evaluate the implications of interleukin-31 (IL-31) in the pathogenesis of chronic spontaneous urticaria (CSU) and to assess the differences that occur between its serum values compared to controls. Additionally, the serum IL-31 levels were measured alongside other clinical and paraclinical parameters that were identified in the patients to understand its immunological importance in this skin disease and to determine if it could potentially serve as a therapeutic target in CSU in the future. (2) Methods: The serum levels of IL-31 were estimated in 50 patients diagnosed with CSU according to the accepted international guidelines. Additionally, 38 controls who had not experienced any episodes of urticaria during their lifetime were included. (3) Results: Significantly elevated serum IL-31 levels were observed in CSU patients compared to the controls (*p* < 0.0001). Although no direct correlations were found between IL-31 and inflammatory markers (erythrocyte sedimentation rate (ESR), C-reactive protein (CRP)), eosinophils, or total immunoglobulins E (IgE), significant differences in IL-31 levels were identified based on CSU severity, quality of life impact, itch intensity, and response to histamine H1 receptor antagonists (H1 antihistamines) (*p* < 0.05 for all). (4) Conclusions: Our findings underscore that IL-31 is not directly associated with general inflammation, eosinophilic response, or atopy in CSU. Nevertheless, its expression is influenced by key disease characteristics: severity, pruritus, and H1 antihistamine response. This investigation provides essential insights into CSU pathogenesis, potentially leading to novel therapeutic interventions. An enhanced understanding of these mechanisms is crucial due to the limitations of current treatment modalities in terms of fully managing CSU symptoms.

## 1. Introduction

Chronic urticaria, characterized by a persistent rash and intense pruritus lasting over six weeks, involves complex pathogenetic mechanisms that remain incompletely understood [1,2]. The roles of mast cells, basophils, and eosinophils in the disease’s pathogenesis are well-known, leading to the release of vasoactive mediators, including histamine, proinflammatory compounds, and newly synthesized cytokines [1,2]. Cytokine-mediated inflammation, particularly alarmins such as interleukin-33 (IL-33), interleukin-25 (IL-25), and thymic stromal lymphopoietin (TSLP), originating from mast cells, play significant roles in inflammation [3,4,5]. Additionally, interleukin-31 (IL-31), which is produced by various immune cells including T helper 2 (Th2) cells, mast cells, macrophages, and dendritic cells, has some importance in pruritus and inflammatory responses [6,7,8].

Current therapies for chronic spontaneous urticaria (CSU) include second-generation H1 antihistamines, omalizumab, ciclosporin, and corticotherapy [1]. However, many patients continue to suffer from uncontrolled symptoms, necessitating the exploration of novel biological treatments [6]. Notably, CSU shares intense pruritus in common with other autoimmune and allergic skin diseases, such as bullous pemphigoid (BP), psoriasis, and atopic dermatitis (AD) [7,8,9]. While histamine and neuropeptides have traditionally been linked to pruritus, recent evidence highlights IL-31, known as the pruritogenic cytokine, as a pivotal factor in the itch mechanism [7,8,9]. Initially attributed to activated T helper cells, recent studies reveal eosinophils as significant IL-31 sources, implying their role in mediating itch severity [2,10,11,12]. IL-31, besides inducing itching, exerts potential immunomodulatory functions in supporting Th2-type immunity, contributing to the autoimmune diseases associated with IgE, including chronic urticaria [9,10,13,14,15]. Recognizing IL-31’s implication in pruritus and its relevance in AD and other allergic conditions has fostered its consideration as a therapeutic target [7,8,10]. However, the precise implications of IL-31 in CSU, encompassing disease severity, itch intensity, and quality of life impact, remain incompletely elucidated. To address this gap, our study aims to unravel the correlations between serum IL-31 levels and diverse clinical and paraclinical parameters in CSU patients. By unveiling these associations, our investigation seeks to provide novel insights into CSU’s underlying mechanisms and potentially establish IL-31 as a promising therapeutic focus.

Distinguishing itself from prior research centered on IL-31’s role in pruritus, inflammation, and immune modulation, our study specifically delves into IL-31’s involvement within the context of CSU [6,7,8]. Through these intricate associations, our research offers a fresh perspective on IL-31’s significance in CSU and sets the stage for future research directions.

Within this manuscript, we present our findings, highlighting the elevated IL-31 levels found in CSU patients compared to controls. We delve into the correlations between IL-31 and disease severity, itch intensity, and treatment responsiveness. Furthermore, we investigate how IL-31 levels relate to the impact on patients’ quality of life. By illuminating these multifaceted connections, our study enriches our understanding of IL-31’s role in CSU and establishes a foundation for potential therapeutic advancements. The prospect of blocking IL-31 receptors through therapeutic approaches, exemplified by Nemolizumab, emerges as a potential strategy for treating disorders involving IL-31, including CSU [16,17,18].

In summary, this study endeavors to uncover IL-31’s distinctive contribution to CSU, highlighting the novel connections between this cytokine and disease manifestations. With IL-31 as our focal point, we aspire to pave the way for therapeutic interventions that alleviate the burden of CSU for affected individuals.

## 2. Materials and Methods

This research undertakes a retrospective, analytical approach to a study conducted at the Allergology Department of the Regional Institute of Gastroenterology and Hepatology in Cluj-Napoca (IRGH), Romania. The study encompassed 50 patients rigorously diagnosed with CSU in accordance with international consensus guidelines [1]. These guidelines define CSU as the recurrence of a specific maculopapular rash, optionally accompanied by angioedema, manifesting at least twice a week for a period exceeding six weeks [1,2]. To draw a meaningful comparison, a control group was assembled from 38 Institute staff members. Therefore, the inclusion criteria for patients required a diagnosis of CSU in accordance with the referenced guidelines [1]. Notably, patients were required to lack systemic illnesses concurrent with urticaria, such as systemic mastocytosis, Schnitzler syndrome, and urticarial vasculitis, which were considered exclusion criteria for this study. The exclusion criteria for patients also included renal, hepatic, psychiatric, and infectious diseases that may be accompanied by cutaneous eruptions or itching. Conversely, the inclusion criteria for control subjects mandated a complete absence of urticaria episodes throughout their lifetimes. The exclusion criteria for the control group incorporated individuals with a history of acute or chronic urticaria episodes from any etiology, as well as those with systemic conditions characterized by urticaria, wheals, or other cutaneous exanthems, along with pruritus, either individually or in combination, as concurrent symptoms. These systemic conditions encompass renal and hepatic disorders, psychogenic pruritus, other psychiatric conditions, infectious diseases, and malignancies. This study was approved by the Ethics Committee of the “Iuliu Hatieganu” University of Medicine and Pharmacy (UMF), Cluj-Napoca, Romania (AVZ270/10.10.2022) and by the IRGH (12637/11.10.2022). Informed consent was obtained from all participants.

Demographic data and the baseline characteristics of all participants were recorded, including age, sex, and serum IL-31 levels. In patients with CSU, additional data were collected, such as the duration of the disease, the severity of the disease, and the presence or absence of atopy, proven by a positive skin-prick test to environmental allergens. Complementary paraclinical tests were also performed, including a complete blood count, an analysis of inflammatory parameters such as ERS and CRP, coproparasitological examination, and total serum IgE. All laboratory analyses were performed at the hospital’s central laboratory.

Venous blood samples were taken from all these participants and the samples were centrifuged. The serum was later frozen and kept at −80 degrees Celsius. IL-31 concentrations in the serum were assessed using a commercially available enzyme-linked immunosorbent assay (ELISA), following the manufacturer’s instructions (Human IL-31 ELISA Kit, BioLegend, 8999 BioLegend Way, San Diego, CA 92121, United States).

The urticaria activity score over 7 days (UAS7) is an internationally authorized tool used to assess the severity of CSU. It combines the scores for hives and itch severity over a continuous period of 7 days, with scores ranging from 0 to 42. The hives score ranges from 0 to 3, indicating the level of hives: 0 represents no hives, 1 indicates mild hives (fewer than 20 hives in 24 h), 2 corresponds to moderate hives (20–50 hives in 24 h), and 3 signifies severe hives (more than 50 hives in 24 h or a large confluent area of hives). Likewise, the itch severity score ranges from 0 to 3, reflecting the intensity of itchiness: 0 signifies no itch, 1 indicates a mild itch (present but not bothersome), 2 represents a moderate itch (troublesome but not disrupting normal daily activities or sleep), and 3 indicates a severe itch (interfering with normal daily activities or sleep). Based on the UAS7 scores, the patients’ chronic spontaneous urticaria severity is categorized into three disease activity states: scores from 0 to 15 are classified as mild, scores from 16 to 27 are categorized as moderate, and scores from 28 to 42 are considered severe [19,20].

Additionally, the weekly itch severity is also grouped into three levels: scores from 0 to 7 are considered mild, scores from 8 to 14 are classified as moderate, and scores from 15 to 21 are categorized as severe.

The dermatology life quality index (DLQI) is a widely used tool to assess how CSU affects a person’s quality of life. It consists of ten questions, each scored from 0 to 3, with higher scores indicating a more significant impact. The total DLQI score can range from 0 to 30, and the scoring breakdown is as follows: 0 to 1—no impact on quality of life, 2 to 5—small impact, 6 to 10—moderate impact, 11 to 20—very large impact, and 21 to 30—extremely large impact [21,22].

### Statistical Analysis

The data were reported as the median, along with the interquartile range, unless specifically mentioned otherwise. To assess the normality of the data distribution, the Shapiro–Wilk test was conducted. For comparisons between the study groups, the Mann–Whitney U test or the Kruskal–Wallis test was utilized, depending on the nature of the data. The Mann–Whitney U test was chosen when comparing two independent groups, such as the serum IL-31 levels between healthy controls and urticaria patients. This test is suitable for non-normally distributed continuous data and allows us to assess whether there are significant differences in the central tendencies of the two groups. To provide a comprehensive understanding of the practical significance of these findings, we calculated the effect size measures. Effect size measures help elucidate the magnitude of observed differences beyond statistical significance. Thus, in our analysis, we included effect size measures using the Rosenthal coefficient (R) for the Mann–Whitney test, which quantifies the practical significance of the differences observed between these two groups. We calculated this coefficient manually, based on the U statistic and sample sizes. To avoid confusion between the traditional notation “r” for the Rosenthal coefficient and the “r” notation for the Pearson correlation coefficient, we have chosen to represent the effect size coefficient, Rosenthal’s, as “R” in our study. Conversely, the Kruskal–Wallis test was employed when comparing IL-31 levels among more than two independent groups or when dealing with ordinal data. This test extends the Mann–Whitney U test to multiple groups and examines whether there are significant differences in the medians of these groups. In our study, it was used to compare IL-31 levels among patients with different disease severities. To assess the practical significance of the Kruskal–Wallis test results, we calculated the effect size using the epsilon-squared (ε^2^) method. This approach provides a measure of the proportion of variability in the dependent variable that can be attributed to group differences, helping us interpret the practical significance of the findings.

Categorical data were compared using Pearson’s chi-squared test, which is appropriate for analyzing the associations between categorical variables. This test helped us to determine whether there were significant differences in the categorical variables, such as atopic status, between groups. The relationships between different parameters were determined using Spearman’s correlation, a non-parametric measure suitable for assessing non-linear associations between variables. This analysis enabled us to examine the strength and direction of correlations between IL-31 levels and other clinical parameters. To investigate whether IL-31 could discriminate between healthy controls and urticaria patients, the receiver operating characteristics (ROC) curve for IL-31 was analyzed, and the area under the curve (AUC) was calculated. This analysis allowed us to assess the diagnostic performance of IL-31 in differentiating between the two groups. GraphPad Prism 9.0 software (GraphPad Software Inc., San Diego, CA, USA) was used for conducting the statistical analyses and generating graphs. Statistical significance was set at a *p*-value of <0.05, indicating that results with *p*-values lower than this threshold were considered statistically significant. The choice of these specific tests and methods aimed to comprehensively analyze the relationships and differences in IL-31 levels and the associated clinical parameters within the context of CSU.

## 3. Results

### 3.1. Clinical Data

We meticulously collected the foundational clinical characteristics of the study subjects. A comprehensive synthesis of these characteristics is presented in Table 1 for detailed review [Table 1].

### 3.2. Serum IL-31 Levels in UCS Patients and Controls

**Figure 1:**
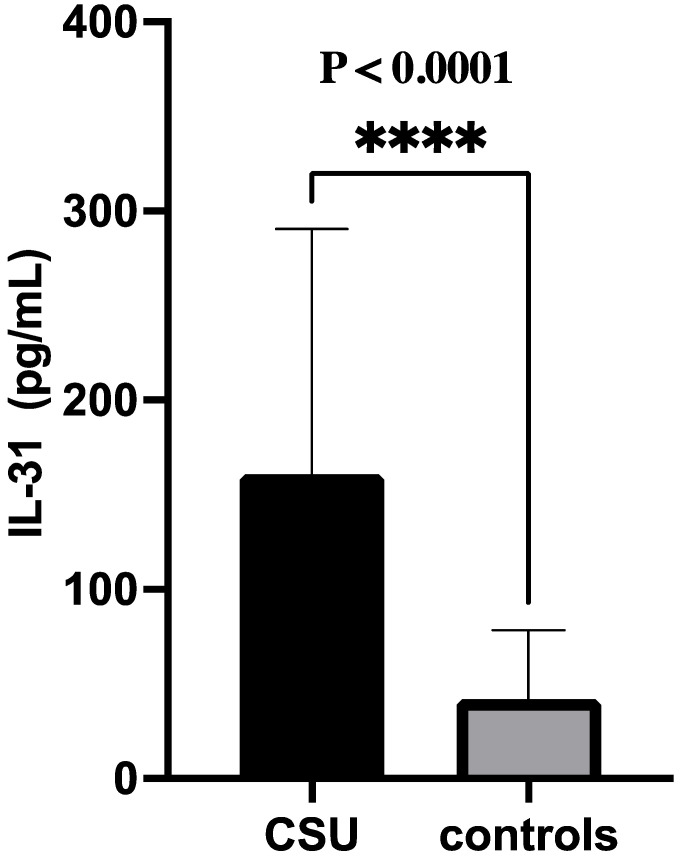
IL-31 serum levels in CSU patients versus (vs.) controls; IL-31—interleukin 31, CSU—chronic spontaneous urticaria, and **** = *p* < 0.0001.

The statistical analysis showed a highly significant difference in serum IL-31 levels between patients and controls, with IL-31 being significantly higher in patients (*p* < 0.0001). The median IL-31 level in patients was 121.5, while in the controls, it was 29.21 (Figure 1). We utilized the Rosenthal coefficient (R) as an effect size measure in conjunction with the Mann–Whitney U test to evaluate the clinical significance of the IL-31 level differences. The computed Cohen’s r value was approximately 0.485, indicating a moderate degree of practical relevance. In essence, this suggests that the observed increase in IL-31 levels among patients compared to controls transcends statistical significance, underscoring its substantial clinical implications.

**Figure 2:**
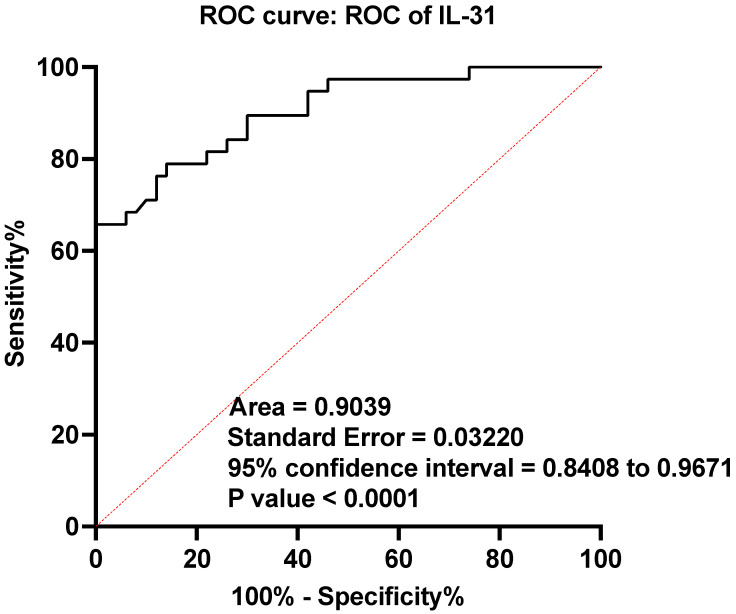
Receiver operating characteristics (ROC) curve of IL-31 in CSU compared with healthy controls; IL-31—interleukin 31, CSU—chronic spontaneous urticaria, *p* < 0.0001, AUC—area under the curve = 0.9039, and SE—standard error = 0.03220.

In our study, ROC analysis was also performed to evaluate the levels of IL-31 between patients with CSU and the control subjects. The AUC was calculated to be 0.9039, with a standard error (SE) of 0.03220 and a 95% confidence interval ranging from 0.8408 to 0.9671 (Figure 2).

### 3.3. Serum IL-31 Levels in Relation to Other Paraclinical Parameters: Eosinophils, Inflammation Markers, and Total IgE Serum Level

**Figure 3:**
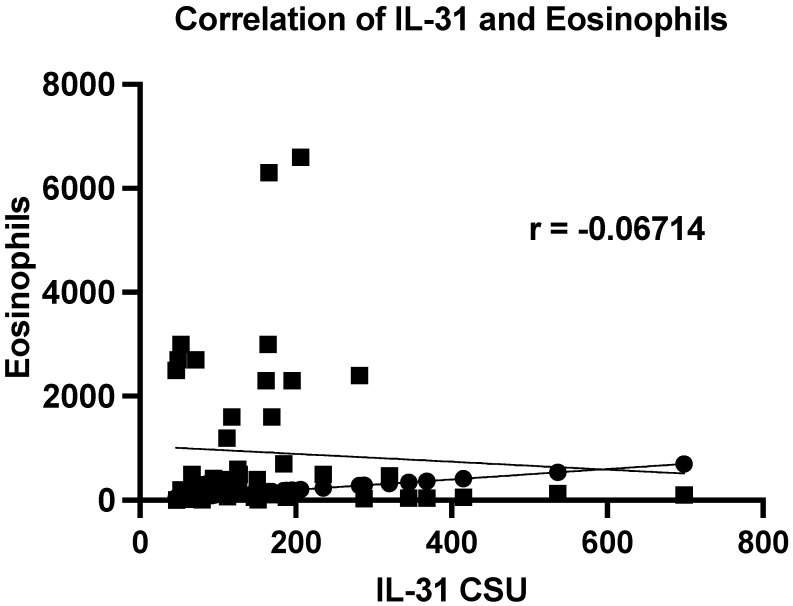
Correlation of serum IL-31 and eosinophils. IL-31—interleukin 31, CSU—chronic spontaneous urticaria, *p* = 0.6432; r—the Pearson correlation coefficient for the serum level of IL-31 and serum eosinophils is −0.06714 (r = −0.06714).

There were no significant correlations between IL-31 levels and other paraclinical parameters, such as serum eosinophils, or inflammatory markers, such as ESR or CRP, as well as total IgE levels. The Pearson correlation coefficient (r) for the serum level of IL-31 and serum eosinophils is −0.06714, indicating a weak negative correlation between the two variables. However, this correlation was not statistically significant (*p* = 0.6432) as the *p*-value was greater than 0.05. The analysis was based on 50 XY pairs, and the results suggested that there was no significant relationship between serum interleukin-31 levels and serum eosinophil levels in patients (Figure 3). Using the same statistical analyses, correlations between IL-31 and ESR, CRP, and total IgE levels were investigated, but no statistically significant associations were found between them.

### 3.4. Serum IL-31 Levels in Relation to Atopy

**Figure 4:**
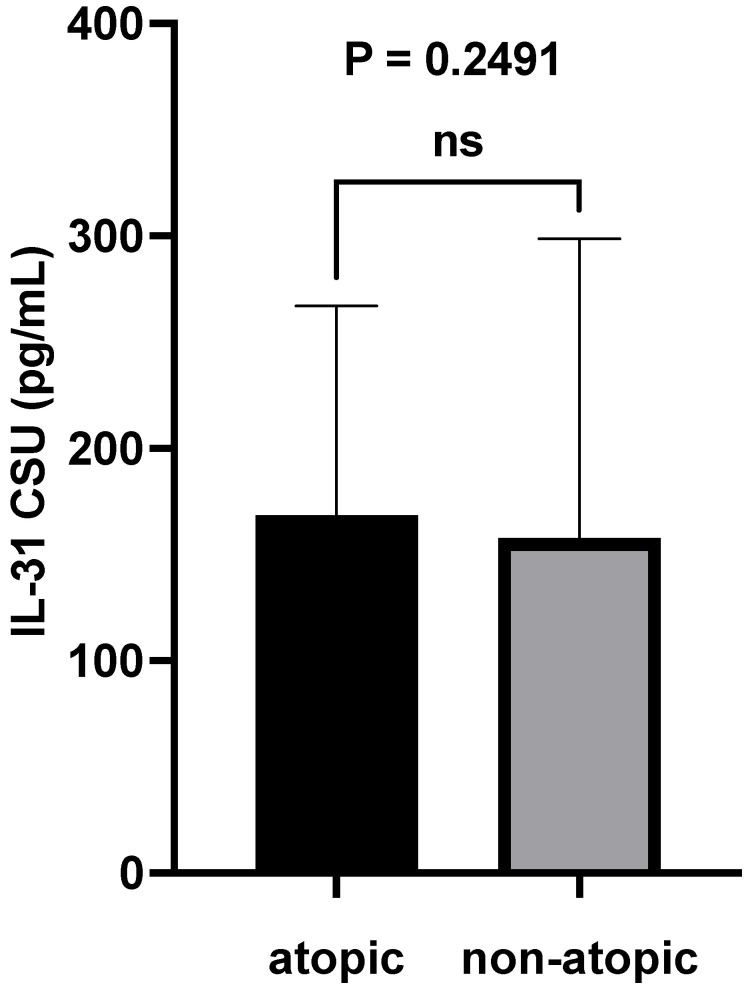
IL-31 serum levels in atopic vs. non-atopic patients; IL-31—interleukin 31, CSU—chronic spontaneous urticaria, ns—non-significant, and *p* = 0.2491.

Another objective of our study was to examine potential variations in serum IL-31 levels in relation to atopic status, specifically contrasting these levels between atopic and non-atopic CSU patients. Statistical analysis conducted using the Mann–Whitney test yielded a non-significant *p*-value of 0.2491, thus indicating that there was no statistically significant difference in serum IL-31 levels between these subgroups (Figure 4). The Rosenthal coefficient (R) for the comparison between non-atopic and atopic groups regarding IL-31 levels is approximately −0.214. This result suggests a small or negligible effect. In other words, the observed difference between the two groups in terms of IL-31 levels is not clinically or practically significant since the coefficient is close to zero.

### 3.5. Serum IL-31 Levels in Relation to Clinical Tools

#### 3.5.1. Serum IL-31 Levels in Relation to UAS7

**Figure 5:**
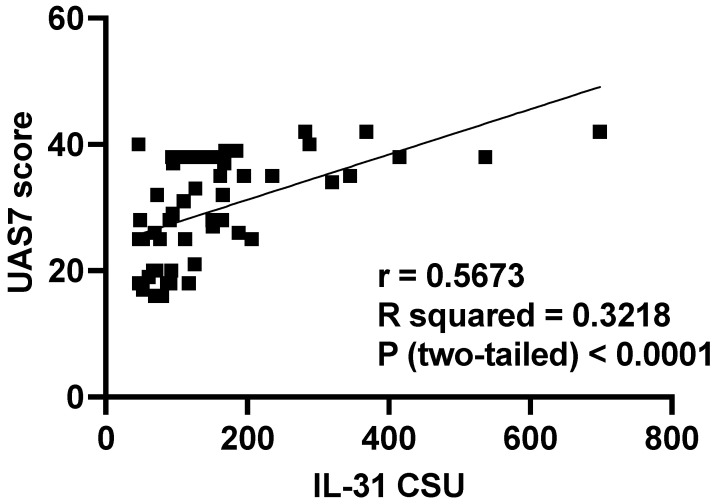
Serum IL-31 levels in patients with moderate vs. severe forms of CSU, according to UAS7; IL-31—interleukin 31, CSU—chronic spontaneous urticaria, UAS7—urticaria activity score per 7 days, - the Pearson correlation coefficient—r = 0.5673, and *p* < 0.0001.

**Figure 6:**
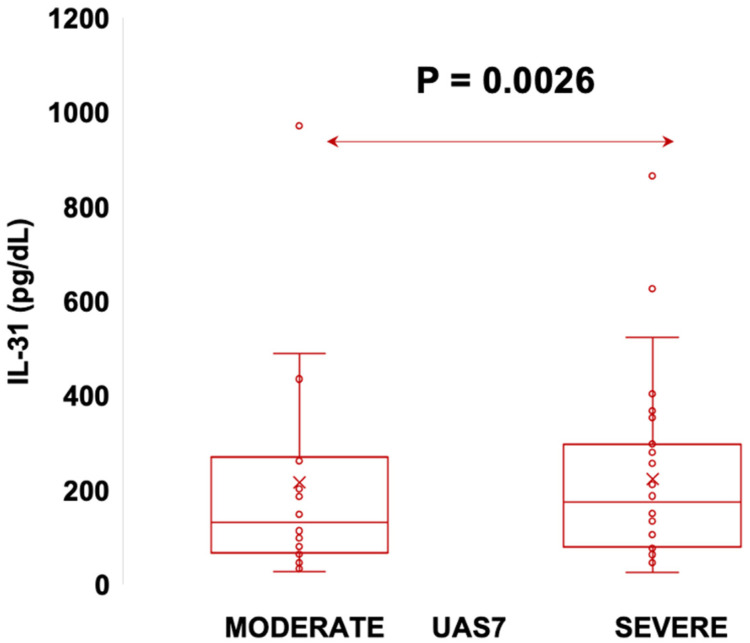
The Mann–Whitney U test, showing the level of serum IgE in moderate and severe forms of CSU, where *p* = 0.0026; IL-31—interleukin 31, CSU—chronic spontaneous urticaria, UAS7—urticaria activity score per 7 days, and *p* = 0.0026.

According to UAS7, which, as explained in materials and methods, divides patients into three categories of disease severity, our patients fell into the moderate and severe forms, with no patient having the mild form of the disease. Thus, we divided the patients into two categories: moderate and severe, and evaluated whether there are differences in IL-31 levels between them. The examination of statistical relationships revealed a significant positive correlation (r = 0.5673, *p* < 0.0001) between IL-31 serum levels in CSU patients and UAS7 scores, underscoring the finding that individuals with higher UAS7 scores exhibited elevated IL-31 levels (Figure 5). Furthermore, the investigation encompassed a comparison of IL-31 levels within distinct disease severity groups using the Mann–Whitney U test. For patients with moderate disease, the mean IL-31 level stood at 106 (standard deviation 42), with a median of 94 (range: 80 to 127). Conversely, patients with severe disease exhibited a mean IL-31 level of 200 (standard deviation 156), accompanied by a median of 163 (range: 77 to 282). The utilization of the Mann–Whitney U test demonstrated a significant differentiation between these two groups (*p* = 0.0026). Specifically, IL-31 levels were notably higher in patients grappling with severe disease compared to those with moderate disease presentation (Figure 6). The Rosenthal coefficient (R) for the comparison between the “moderate form” and “severe form” groups in terms of IL-31 levels was approximately 0.327. This result suggests a moderate effect size. It indicates that there is a moderate practical significance associated with the difference in IL-31 levels between these two groups. In other words, the observed difference in IL-31 levels is not only statistically significant but also carries meaningful clinical implications. Notably, the analysis incorporated data from 21 patients with a moderate disease form and 29 patients with a severe manifestation of the condition.

#### 3.5.2. Serum IL-31 Levels in Relation to the Severity of the Pruritus

**Figure 7:**
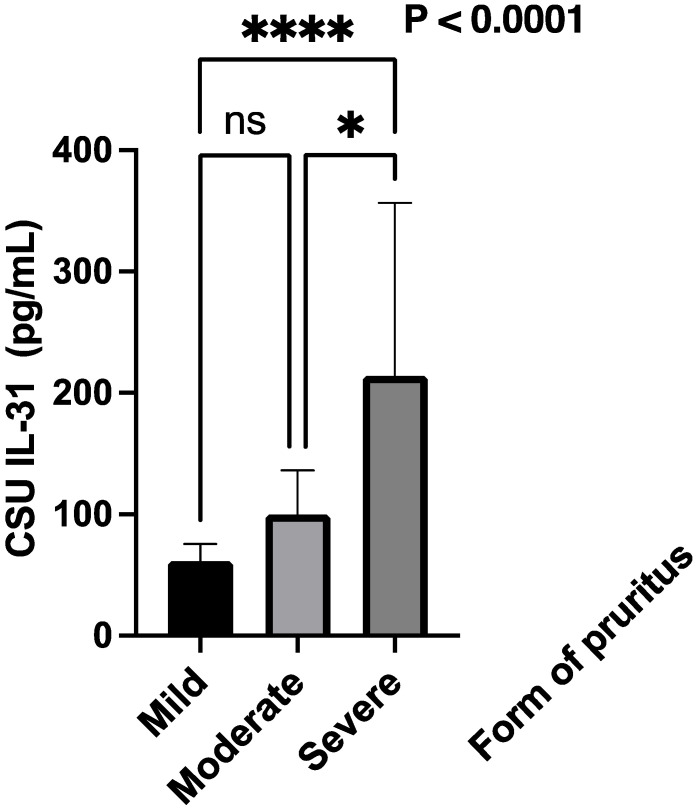
Serum IL-31 levels and the forms of pruritus quantified by the intensity of itch; IL-31—interleukin 31, CSU—chronic spontaneous urticaria, ns—non-significant, and **** = *p* < 0.0001, * = *p* < 0.05.

Additionally, as shown in the second subtitle of this paper, the severity of itch was also quantified separately by summing the scores over 7 days, and patients were divided into three categories, based on the intensity of the pruritus. Scores from 0 to 7 were considered mild, scores from 8 to 14 were classified as moderate, and scores from 15 to 21 were categorized as severe (Figure 7).

**Figure 8:**
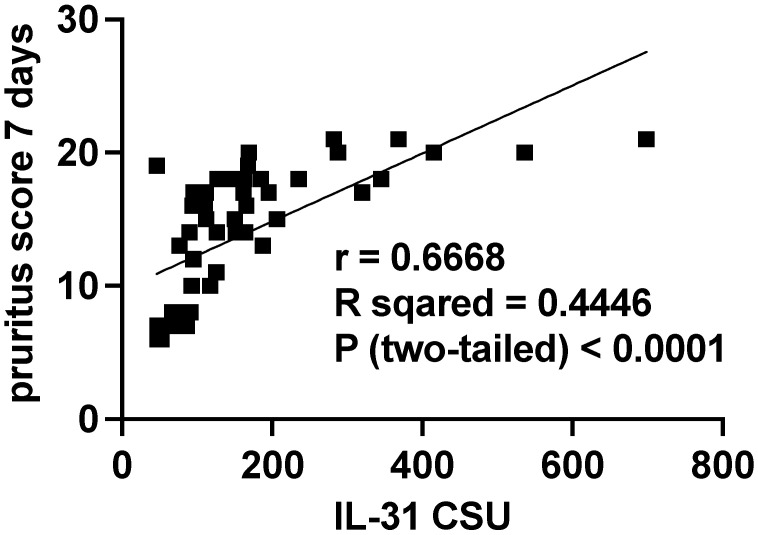
Correlation of serum IL-31 levels in CSU patients and pruritus score per 7 days; IL-31—interleukin 31, CSU—chronic spontaneous urticaria, *p* < 0.0001, and the Pearson correlation coefficient—r = 0.6668.

The statistical analysis used the Kruskal–Wallis test to examine the association between different forms of pruritus. The test resulted in a highly significant *p*-value of less than 0.0001, indicating that there were statistically significant differences between the groups with different forms of pruritus. The *p*-value summary is denoted by ****, confirming the high significance of the result. The test also revealed that the medians of the pruritus scores vary significantly (*p* < 0.05) among the three groups. The Kruskal–Wallis statistic value is 26.79, which further supports the evidence of significant differences in pruritus scores among the groups (Figure 8). The effect-size epsilon-squared (ε^2^) value for the Kruskal–Wallis test using the epsilon-squared method is approximately 0.149. This suggests a moderate effect size, indicating that around 14.9% of the variability in the dependent variable can be attributed to the differences between the groups. In other words, there are meaningful differences among the groups in our study.

#### 3.5.3. Serum IL-31 Levels in Relation to DLQI

**Figure 9:**
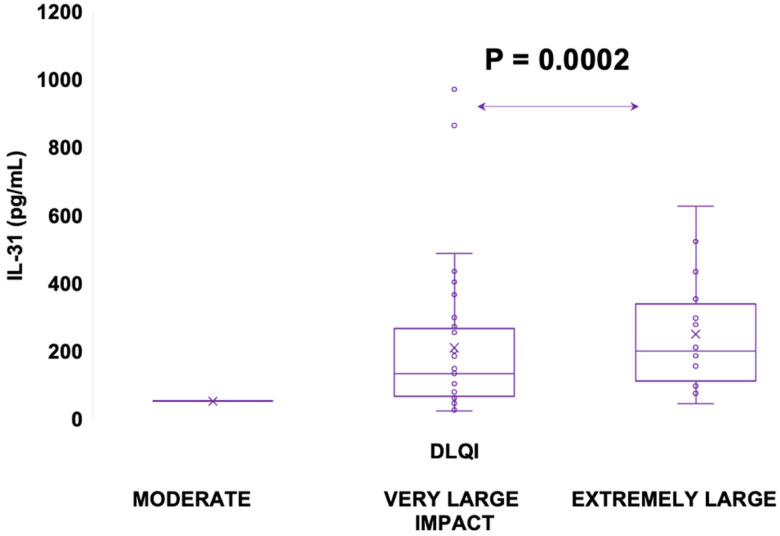
Serum IL-31 levels in CSU patients experiencing a moderate impact, very large impact, and extremely large impact on quality of life, according to the DLQI; IL-31—interleukin 31, CSU—chronic spontaneous urticaria, DLQI—dermatology life quality index, and *p* = 0.0002.

**Figure 10:**
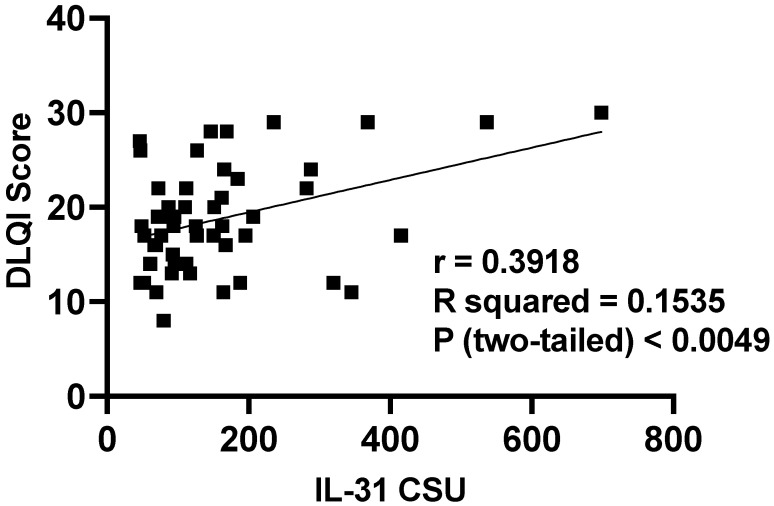
Correlation of serum IL-31 levels in CSU patients and DLQI score; IL-31—interleukin 31, CSU—chronic spontaneous urticaria, DLQI—dermatology life quality index, *p* < 0.0049, and the Pearson correlation coefficient—r = 0.3918.

The impact of CSU on patients’ quality of life was assessed using the dermatology life quality index (DLQI), as explained in the Materials and Methods (Section 2). The results revealed that patients in the “very important impact” group (n = 33) had a mean IL-31 level of 104 (SD = 47), with a median of 93, ranging from 70 to 127 pg/mL. On the other hand, the “extremely important impact” group (n = 16) exhibited a significantly higher mean IL-31 level of 276 (SD = 170) with a median of 244, ranging from 160 to 351 pg/mL. Additionally, it is worth noting that there was one patient with a “moderate” impact on quality of life, as per the DLQI, who had an IL-31 level of 79.635 pg/mL. These findings suggest that higher IL-31 levels may be associated with a more pronounced impact on the quality of life in patients with the disease (Figure 9 and Figure 10).

### 3.6. Serum IL-31 Levels in Relation to Response to Antihistamines AH1

**Figure 11:**
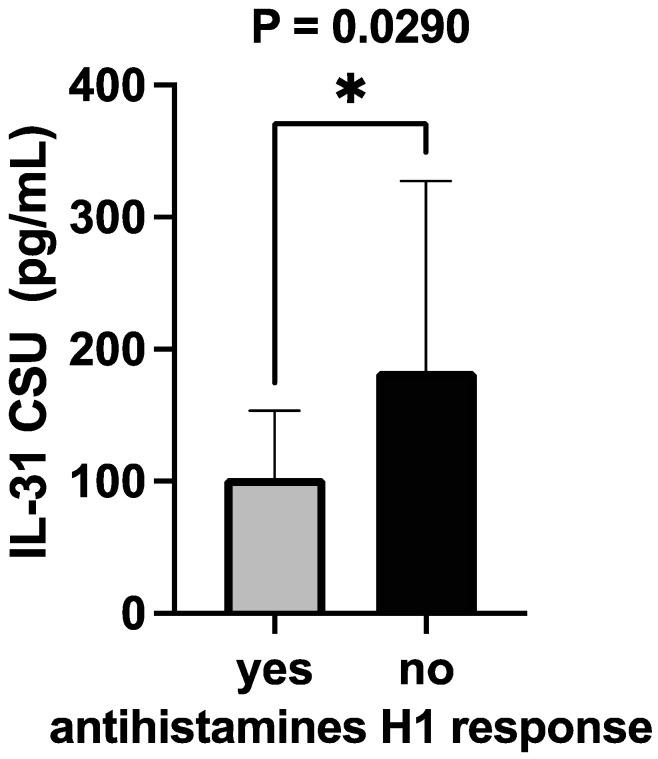
CSU patients’ serum IL-31 levels and the response to antihistamines H1; IL-31—interleukin 31, CSU—chronic spontaneous urticaria, antihistamines H1—histamine H1 receptor antagonists, and * *p* = 0.0290.

The IL-31 levels in CSU patients and their response to the maximum recommended dose of second-generation h1 antihistamines [1] were compared, dividing the patients into two groups, as follows: those who responded to AH1—“YES”; those who did not respond to AH1 requiring the following therapeutic steps—“NO”. The Mann–Whitney test resulted in a *p*-value of 0.0290, indicating a statistically significant difference in the treatment response between the two groups. The “*p*-value summary” denoted by one asterisk (*) confirms a significance at the alpha level of 0.05. The test used a two-tailed *p*-value, considering both higher and lower values. The sum of ranks in the “YES” and “NO” groups was 256.5 and 1019, respectively, with a Mann–Whitney U value of 151.5. The difference between the medians of the two groups was 46.06 (actual difference) or 43.56 (the Hodges–Lehmann estimate). This suggests that the group with a negative response to the treatment (“NO”) had a higher median value of serum IL-31 levels (136.9) compared to the group with a positive response (“YES”) with a median of 90.86 (Figure 11).

We obtained an effect size coefficient, Rosenthal’s coefficient (R) of approximately 0.295. This value indicates a moderate level of practical significance. In essence, it signifies that the difference in response to AH between the “YES” and “NO” groups is not only statistically significant but also carries meaningful clinical implications. This means that beyond the statistical significance observed in our Mann–Whitney test, the practical importance of the difference in AH responses between these two groups is underscored by the calculated effect size.

## 4. Discussion

CSU is a debilitating skin disease that has a considerable impact on the daily life of affected patients, not only through the characteristic lesions but especially through itching. It affects approximately 1% of the global population and represents a substantial socioeconomic burden, with absenteeism from the workplace and high costs for the necessary treatments [23,24,25,26]. Thus, there is an urgent need to better understand the pathogenesis of CSU to develop improved diagnostic and treatment approaches [2,6,27,28]. Despite this need, there has been less focus on pruritus in the specialized literature that studies chronic urticaria, the symptom being more pathognomonic for atopic dermatitis, which is why IL-31 was also studied further in the context of this condition [7,9,29]. Based on these considerations, our study aimed to investigate the correlation between the cytokine attributed to pruritus and the severity scores of the urticaria, the intensity of the itch, and the impact on the quality of life of affected patients. Our findings revealed a significant association between IL-31 levels in CSU patients and pruritus intensity, disease severity, and quality of life. Additionally, we observed that patients who responded to the first steps of treatment, up to the maximum dose of H1 antihistamines, had lower serum levels of IL-31 than those who did not respond to these drugs and required other therapeutic steps. Among these therapeutic options, anti-IL-31 therapy could also be considered, as has been considered for other itchy skin conditions [7,9,29].

IL-31 plays a significant role in cutaneous inflammation and has been extensively studied for its involvement in tissue homeostasis, inflammation, immune defense, neuroimmune circuits, and pruritus [7,29,30,31,32,33]. It belongs to the IL-6-derived cytokine family and is associated with proinflammatory characteristics. While its physiological function is not fully understood, it has been implicated in various inflammatory disorders in humans, such as AD, inflammatory bowel disease, and asthma [9,16,17,29,30,31,32]. IL-31 is produced by TH2 cells and immature dendritic cells, activating neurons and keratinocytes through its receptors, IL31RA/OSMRβ. The intradermal application of IL-31 induces itching, further illustrating its ability to activate target neurons [34,35]. In our study, we found significantly increased levels of IL-31 in patients with CSU compared to healthy controls, which is consistent with observations from other studies [4,5,30]. We also computed the effect size coefficients, which revealed a moderate to large level of significance. In essence, the statistical analysis demonstrated not only a highly significant difference in serum IL-31 levels between patients and controls but also conveyed that this difference holds practical importance in the context of our study. It highlights the potential clinical relevance of elevated IL-31 levels in patients, which may warrant further investigation and consideration in the management of CSU.

We also conducted an ROC analysis to assess the discriminatory ability of IL-31 in distinguishing between individuals with CSU and those without the condition (control subjects). The AUC value that we obtained was 0.9039. This value indicates a high degree of accuracy in distinguishing between the two groups using the IL-31 parameter. The analysis showed that IL-31 is quite effective in making this distinction, with a high degree of certainty. The numbers we provided give us confidence in the accuracy and reliability of our results. This finding supports the inclusion of urticaria in the category of pruritogenic inflammatory skin diseases, along with the others mentioned above.

During the course of our study, we meticulously scrutinized the foundational clinical characteristics of both the CSU patient group and the carefully matched control cohort. Strikingly, no statistically significant disparities emerged in these pivotal attributes, such as age and sex, underscoring the meticulous selection process that aimed to eliminate potential confounders arising from these fundamental demographics. Surprisingly, we also noticed the absence of correlations between the serum levels of IL-31 and eosinophils in our patients, despite prior theoretical assumptions suggesting an association between IL-31 and eosinophils [2,10,11,12]. This discrepancy between IL-31 and eosinophils suggests that while IL-31 may contribute to certain aspects of allergic inflammation, its role might not be as straightforward as previously assumed. Intriguingly, our findings regarding serum IL-31 levels in relation to atopic status present a complex picture that challenges our initial expectations. While previous research has suggested a potential link between IL-31 and atopic conditions [9,16,17,29,30,31,32,35,36] such as AD, the non-significant difference in IL-31 levels between atopic and non-atopic CSU patients, as indicated by the Mann–Whitney test (*p*-value = 0.2491), adds a layer of complexity to our understanding. Furthermore, no significant correlations were identified between non-specific inflammatory markers, such as ESR and CRP, and the serum levels of IL-31. These findings strengthen the notion that IL-31 may not primarily function as a non-specific pro-inflammatory cytokine. Rather, it appears to exhibit distinct characteristics that are intricately connected to the pruritogenic mechanism, as indicated by the observed positive correlations. These correlations, as detailed in the results section and elaborated upon in the subsequent paragraphs of this discussion, underscore the connections we identified between serum IL-31 levels and the specific clinical impacts of pruritus, assessed through the presented scoring systems.

We observed significant correlations between the level of IL-31 and the severity of CSU, as quantified through the UAS7 assessment. These findings align with previous investigations that have also reported associations between IL-31 serum levels and disease severity in patients suffering from CSU [4], atopic dermatitis [36], and uremic pruritus [37]. Notably, a relatively recent study from 2020 demonstrated contrasting results since it found no correlations between IL-31 levels in CSU and psoriasis patients with varying degrees of disease severity, as evaluated by UAS7 and psoriasis area and severity index (PASI) scores, respectively [5]. The correlations observed in our study, corroborating previous research, bolster the prominence of IL-31 in the realm of pruritic skin ailments, particularly CSU. The robust associations between IL-31 and clinical severity scores underscore the significance of this cytokine in the complex tapestry of pruritic pathophysiology. The noteworthy discordant findings in the context of psoriasis and CSU severity further emphasize the need for comprehensive investigations to unravel the multifaceted role of IL-31 in diverse pruritic conditions. It is evident that our findings, along with other pertinent research, lay the groundwork for a deeper understanding of IL-31’s involvement in pruritic skin disorders, particularly CSU. The substantial correlations uncovered in this study contribute to the growing body of evidence highlighting IL-31’s potential as a key player in pruritus etiology and severity. Nonetheless, further exploration and nuanced studies are essential to elucidate the exact mechanisms that underlie the observed correlations, thereby paving the way for potential therapeutic interventions targeting IL-31 to ameliorate pruritus and enhance the quality of life for affected individuals.

In line with contemporary investigations, other studies have emphasized the significance of this cytokine in the pathogenesis of various conditions, including autoimmune skin diseases, atopic dermatitis, and other atopic skin inflammation, as well as in its impact on the quality of life of patients afflicted by these conditions [7,9,29]. Following the lead of these studies, we aimed to investigate whether there exists a correlation between serum IL-31 levels and the impact on the lives of patients with CSU through the utilization of the DLQI score. Thus, we have demonstrated that IL-31 levels in CSU exhibit a direct proportionality with the impact on patients’ quality of life. This finding aligns with the outcomes reported by authors in studies referenced earlier [7,9,29], which similarly encompass conditions marked by pruritus, elevated serum IL-31 levels, and a substantial impact on quality of life. Collectively, these observations underscore a salient association between serum IL-31 levels and their implications for individuals grappling with pruritic disorders. Nevertheless, to solidify and enhance the comprehensiveness of the relationship between IL-31 levels and their impact on quality of life, further investigations involving a larger cohort are imperative.

Past investigations have unveiled alterations in IL-31 levels following targeted therapeutic interventions. For instance, in individuals afflicted by psoriasis, levels of IL-31 in serum exhibited a significant reduction following exposure to narrowband ultraviolet radiation [38]. Furthermore, the efficacious administration of omalizumab to patients grappling with CSU led to noteworthy declines in serum IL-31 concentrations [39]. Additionally, a study involving CSU patients who displayed positive responses to omalizumab elucidated a substantial correlation linking ameliorated clinical symptoms with diminished IL-31-secreting T cells [40]. Although the present study did not explicitly assay IL-31 levels after specific interventions, it was observed that patients who were unresponsive to antihistamine treatment during the initial treatment stages exhibited elevated IL-31 values. In other words, those who solely responded to H1 antihistamines, the primary treatment tier in CSU, demonstrated lower serum IL-31 levels compared to those requiring more intricate therapeutic regimens. These findings, strengthened by effect size coefficients, emphasize the potential clinical relevance of the observed disparities in antihistamine responses and call for further exploration and consideration in clinical practice. Subsequent investigations aimed at quantifying IL-31 levels contingent on diverse treatment modalities and correlating them with cytokine profiles warrant consideration. Such endeavors hold the potential to unravel the nuanced implications of IL-31 in the disease’s pathogenesis, progression, and therapeutic responses.

### Strengths and Limitations of the Study

The present investigation encompasses a range of strengths that enhance its scientific significance within the realm of CSU research. By comprehensively exploring the intricate relationships between serum IL-31 levels and various clinical parameters, as well as the nuanced impact on quality-of-life indicators, this study contributes to a deeper understanding of the potential implications of IL-31 in the context of CSU. Moreover, the meticulous examination of multifaceted aspects such as disease severity, itch intensity, and therapeutic responsiveness bolsters the study’s comprehensive nature.

The strategic inclusion of a control group within the study design further substantiates the validity of the findings by allowing for meaningful comparisons against a reference baseline. Employing well-established diagnostic criteria and employing standardized methodologies, the study demonstrates methodological rigor, ensuring the reliability and reproducibility of results. Additionally, the judicious application of diverse statistical analyses adds a layer of robustness to the interpretation of observed associations.

Notwithstanding these strengths, the study does bear certain limitations that warrant acknowledgment. The relatively constrained sample size could potentially limit the extrapolation of findings to broader populations, necessitating circumspection when considering the broader implications. The inherent cross-sectional nature of the study design precludes the establishment of causal relationships between IL-31 levels and the clinical parameters explored. To unveil the temporal dynamics and elucidate potential causal mechanisms, prospective longitudinal investigations are imperative.

The single-center setting, while providing a controlled environment, introduces the possibility of selection bias and may restrict the generalizability of the findings to more heterogeneous populations. Collaborative endeavors encompassing multiple centers could mitigate this limitation and enhance the external validity of the study outcomes.

In conclusion, while this study substantively enriches our understanding of the intricate interplay between IL-31 and CSU, it is important to underscore that the strengths and limitations outlined herein underscore the necessity for continued research. Ongoing endeavors with larger and more diverse cohorts, encompassing various settings and employing longitudinal designs, are poised to refine and substantiate the current findings, culminating in a more nuanced comprehension of the intricate interrelationships governing IL-31’s role in the clinical context of CSU.

## 5. Conclusions

In conclusion, our study offers valuable insights into the multifaceted interactions between IL-31 and CSU, shedding light on the complex role of this cytokine in pruritic skin disorders. The significant associations that we observed between serum IL-31 levels and pruritus intensity, disease severity (UAS7), and quality of life (DLQI) underscore IL-31’s potential as a key player in the pathophysiology of CSU. Moreover, the correlation between lower IL-31 levels and a positive response to initial H1 antihistamine treatment highlights the potential relevance of IL-31 as a therapeutic target.

The intricate web of relationships that we have unveiled underscores the intricate nature of pruritus etiology and severity and supports the need for further investigation into the underlying mechanisms. As IL-31 emerges as a potential biomarker for disease severity and therapeutic response, future studies with larger, more diverse cohorts and prospective longitudinal designs will provide a deeper understanding of IL-31’s temporal dynamics and causal relationships.

While our study advances our understanding of IL-31’s involvement in CSU, it is essential to acknowledge the study’s limitations, such as the sample size and single-center setting, which call for continued research in more diverse settings. Collectively, the findings presented here contribute to the ongoing pursuit of improved diagnostic tools and therapeutic approaches for individuals grappling with the burden of CSU.

## Figures and Tables

**Table 1:** Characteristics of the study participants. Note: F, female; M, male. Age is presented as mean ± standard deviation (SD).

Characteristic	CSU Patients	Controls
Number, n	50	38
Sex (F/M)	36/14	26/12
Age, yrs.	50.14 ± 16.10	44.32 ± 9.23
Atopy (Atopic/Non-atopic)	14/36	10/28

## Data Availability

All data generated or analyzed during this study are included in this article. Further inquiries can be directed to the corresponding author.
